# A comprehensive evaluation of MRI-based radiogenomics and prognosis prediction in glioma

**DOI:** 10.3389/fonc.2025.1679634

**Published:** 2026-01-05

**Authors:** Mehdi Astaraki, Marta Lazzeroni, Iuliana Toma-Dasu

**Affiliations:** 1 Division of Medical Radiation Physics, Department of Physics, Stockholm University, Stockholm, Sweden; 2 Department of Oncology-Pathology, Karolinska Institutet, Stockholm, Sweden; 3 Department of Clinical Science, Intervention and Technology, Karolinska Institutet, Stockholm, Sweden

**Keywords:** brain segmentation, glioma, radiogenomics, radiomics, survival status

## Abstract

**Background and purpose:**

In gliomas, characterization of the molecular landscape plays a critical role in determining prognosis and guiding treatment regimens. Imaging biomarker models hold promise for non-invasive characterization of glioma subtypes. We, comprehensively, assessed the potential of magnetic resonance imaging (MRI) data for predicting the survival status and molecular subtypes of glioma.

**Methods:**

We introduce a novel method for quantifying the spatial distribution of gliomas within brain anatomy. The method measures the volumetric ratio of 32 brain anatomical structures affected by the tumor. This novel feature set was combined with established radiomics to build models for predicting O6-methylguanine-DNA methyltransferase (MGMT) methylation status, isocitrate dehydrogenase (IDH) mutation status, and Overall Survival (OS) time of glioma patients. The performance of these models was evaluated on preoperative MRIs of 1788 subjects from four independent datasets, employing both cross-validation (CV) and cross-dataset evaluation strategies.

**Results:**

The proposed feature set revealed no regular patterns in tumor locations across the brain. Integration of these features with radiomics improved model performance for the three tasks. The best performance, in terms of AUROC, respectively, for CV and cross-data tests were: 0.685 and 0.628 for MGMT status, 0.972 and 0.764 for IDH status, and 0.748 and 0.719 for OS time status.

**Conclusions:**

Our experiments demonstrate the potential of imaging biomarkers for IDH prediction, highlighting the challenges associated with predicting MGMT and OS only from image data. This underscores the need for additional information beyond MRI, for accurate prediction of these prognostic markers.

## 1 Introduction

Gliomas are the most common malignant primary brain tumors in adults. Based on the 2021 World Health Organization (WHO) classification, three main types of gliomas are astrocytoma, oligodendroglioma, and glioblastoma (GBM) (1). Despite advances in treatment regimens, including surgical resection to the maximal safe extent followed by radiation and chemotherapy, the survival rate of GBM patients, the most aggressive type of glioma, remains poor, e.g., the median overall survival (OS) time is less than 15 months (2, 3). Treatment options for glioma subtypes are different (4). In this context, there are two important biomarkers representing different molecular attributes of glioma: (1) O6-methylguanine-DNA methyltransferase (MGMT) promoter methylation status and (2) isocitrate dehydrogenase (IDH) mutation status. Specifically, MGMT methylation status is accepted as one of the leading determinants of prognosis and a potential predictor of response to temozolomide therapy and, therefore, would influence neoadjuvant options before surgery (5). In general, patients with methylated MGMT promoters have longer survival rates than those with unmethylated MGMT promoters. In addition, IDH status indicates the tumor grade, including low-grade diffuse gliomas (IDH-mutated) and GBM (IDH-wildetype) with IDH-mutated tumors have better prognosis. Therefore, inaccurate classification of tumor subtypes negatively impacts patient’s treatment options and can result in limited access to neoadjuvant therapies (6). Stratifications of the glioma subtypes require invasive biopsy procedures with a risk of sampling bias due to the inherent heterogeneity of genetic features (7). Statistics from the cancer genome atlas pilot project show that only 35% of all sampled tissues via biopsy had sufficient tumoral contents for accurate glioma molecular profiling (8). Hence, developing accurate and reliable non-invasive alternative methods would be beneficial for the diagnosis and treatment of glioma patients.

Radiogenomics refers to the study of the correlation between imaging phenotypes and underlying gene expression of cancerous tissues. The ability of Magnetic Resonance Imaging (MRI) to, non-invasively, provide spatial heterogeneity within the tumors on one side, and advances in the field of radiogenomics on the other side, resulted in the development of a variety of imaging biomarkers models for glioma prognosis and subtype classification (9). Several studies showcased the effectiveness of radiomics and/or deep learning (DL) techniques in predicting IDH mutation, MGMT promoter methylation, and survival status of glioma patients (10–12). In a recent comprehensive study, Lost et al. (4), reviewed 85 articles that met their requirements from 1323 preliminary selected articles. They summarized that the reported prediction power, in terms of area under a receiver operating characteristic curve (AUROC) for IDH mutation status lies around 0.88, and for MGMT methylation status, this metric lies around 0.77. However, there exist other studies showing the inability of imaging biomarkers for accurate molecular profiling and prognosis predictions of gliomas (13). As an example, the 2021 RSNA-MICCAI Brain Tumor Segmentation (BraTS) challenge (14) aggregated imaging data from five public repositories as well as a number of private institutions and aimed to, objectively, evaluate the performance of radiomics/DL models for the prediction of MGMT methylation status. Nevertheless, the top-performing models resulted in AUROC values below 0.6 on the test set (15).

In this study, we introduce a new feature set that is capable of capturing the spatial distribution of gliomas within the normal brain anatomy. Then, we conduct a comprehensive analysis to answer the question of whether MRI data can be used for accurate prediction of IDH mutation status, MGMT methylation status, and survival status of gliomas by employing several large-scale MRI datasets.

## 2 Materials and methods

### 2.1 Datasets

Four datasets were examined to investigate the potential of imaging biomarkers models for glioma molecular profiling and OS time prediction.

#### 2.1.1 MICCAI-RSNA 2021 radiogenomics dataset

The Brain Tumor Segmentation (BraTS) community in collaboration with the Medical Image Computing and Computer Assisted Intervention Society (MICCAI) and the Radiological Society of North America (RSNA) held a challenge in 2021 targeting the evaluation of computational algorithms for two independent tasks: (i) glioma segmentation and (ii) glioma molecular characterization in terms of MGMT methylation status (14). The MGMT classification task contains multiparametric MRI (mpMRI) scans for 585 glioma patients categorized into two groups: methylated and unmethylated MGMT.

#### 2.1.2 UPenn-GBM dataset

The University of Pennsylvania Glioblastoma Imaging, Genomics, and Radiomics (UPenn-GBM) dataset incorporates the currently largest publicly available preoperative dataset of 611 patients diagnosed with GBM. This collection includes advanced mpMRIs (structural, diffusion, and perfusion images), the molecular status of IDH mutation and MGMT methylation, as well as OS time (16).

#### 2.1.3 UCSF Glioma MRI dataset

The University of California San Francisco Preoperative Diffuse Glioma MRI (UCSF-PDGM) dataset contains 501 patients diagnosed with diffuse gliomas. The preoperative scans were acquired with 3-T MRI machines following standard protocols for brain tumor imaging and included structural, diffusion, and perfusion volumes. The dataset also includes IDH mutation and MGMT promotor methylation status for WHO grade 3 and 4 gliomas as well as OS time (17).

#### 2.1.4 LUMIERE GBM dataset

LUMIERE is an MRI dataset of 91 GBM patients, which includes both preoperative and selected follow-up studies according to the response assessment of neuro-oncology criteria (RANO). The provided structural mpMRIs were accompanied by supplementary clinical information including IDH mutation and MGMT methylation status, along with OS time (18).

The imaging data for each subject in all the studied datasets incorporate structural mpMRI sequences, including T1-weighted (T1), T1-weighted post-contrast (T1CE), T2-weighted (T2), and T2 Fluid Attenuated Inversion Recovery (T2-FLAIR). The UPenn and UCSF datasets contain additional MRI sequences, such as diffusion and perfusion derivatives. Therefore, to avoid inconsistency in the analysis steps, we employed only the structural MRIs. In addition to the imaging data, the studied datasets already consist of segmentation masks of Gross Tumor Volumes (GTVs) which include enhancing tumor core, necrotic/cystic core, and peritumoral edematous/infiltrated tissue. A summary of the studied datasets is presented in **Table 1**.

**Table 1:** An overview of the studied datasets. The number of available subjects for each dataset, task, and target label is presented inside the parenthesis.

Dataset (# subject)	Survival (# subject)	MGMT (# subject)	IDH (# subject)
MICCAI-RSNA (585)	None	methylated (307) unmethylated (278)	None
Upenn (611)	days count (452) unknown (159)	methylated (140) unmethylated (177) unknown (294)	mutated (16) wildtype (499) NOS/NEC^1^ (96)
UCSF (501)	days count (500) unknown (1)	methylated (302) unmethylated (114) unknown (85)	mutated (103) wildtype (398)
LUMIERE (91)	weeks count (86) unknown (5)	methylated (37) unmethylated (43) unknown (11)	mutated (1) wildtype (57) negative (10)
Total (1788)	1038	1398	1180

### 2.2 Data preparation and preprocessing

The provided MICCAI-RSNA, UPenn, and UCSF datasets were already preprocessed, i.e., coregistered, resampled, and aligned to the SRI24 atlas (19), as well as skull-stripped. No further preprocessing steps were applied to these images. Accordingly, the same protocols were applied to the preoperative scans in the LUMIERE dataset. Specifically, the highest resolution scan among MRI sequences of each subject was determined and used as a reference to resample other sequences in order to bring the patient’s MRI sequences into a unique space. Then, the T1 sequence was employed as the moving image in registration to the SRI24 atlas. The obtained deformation field was utilized to warp the remaining sequences.

#### 2.2.1 Definition of class labels

The following strategies were applied to the provided clinical information to define the target labels for model development. For the task of OS time prediction, the median survival time among each dataset was chosen as a threshold to categorize the OS status into a binary class (short-term vs. long-term survivors). The MGMT status prediction is defined as a binary task (methylated vs. unmethylated). Finally, the IDH status prediction was determined as a binary problem to classify IDH-wildtype gliomas from the rest.

### 2.3 Spatial distribution pattern

In this section, we introduce a new feature set, *spatial distribution pattern*, which aims at quantifying the anatomical distribution of gliomas in brain structures by segmenting the MRI data into constituent sub-structures.

Parcellation of brain anatomy in MRIs is a fundamental technique in neuroscience communities to study brain organization and functionality (20). However, the presence of major anomalies such as brain tumors changes the appearance of healthy structures and deforms the surrounding tissues. To delineate the anatomical regions of the brain despite the presence of gliomas, we propose the following strategy.

First, we utilized the SynthSeg model to segment the brain structures in T1 images into 32 constituent anatomical regions (mask_Synth_). **Supplementary Table 1** shows the names of anatomical structures with corresponding class labels. SynthSeg is an advanced out-of-the-box DL model developed for brain segmentation in MRIs (21, 22). This model is trained on synthetic image data, which were randomly sampled from a generative model that was conditioned on the segmentation masks and exposed to a different combination of contrast, resolution, morphology, artifacts, and noise. Specifically, the generation process begins with a 3D anatomical label map, which is first deformed using a combination of random affine and non-linear transformations to simulate morphological variability. To generate the image intensities, a Gaussian Mixture Model (GMM) conditioned on the deformed labels is sampled at every voxel. The means and variances of the GMM are drawn from uniform distributions at each training step, fully randomizing the contrast and noise levels. The resulting image is further corrupted by a simulated bias field and a random Gamma transform to augment intensity distributions. Finally, to ensure robustness against resolution differences, the model simulates slice thickness via Gaussian blurring and slice spacing via downsampling, before resampling the image to the target resolution. Therefore, the model can robustly segment brain structures in real MRIs of any contrast and resolution. While this model can accurately segment anatomical subregions even in the presence of small lesions or small-field deformations, it cannot robustly determine the anatomical structures centered/around the gliomas. Therefore, the main source of failure in the segmentations resulting from SythSeg is concentrated on the glioma regions. We, temporarily, masked out these tumoral regions from the obtained segmentations by using the Gross Tumor Volume (GTV) masks (mask_Synth-NoTumor_).

**Figure 1:**
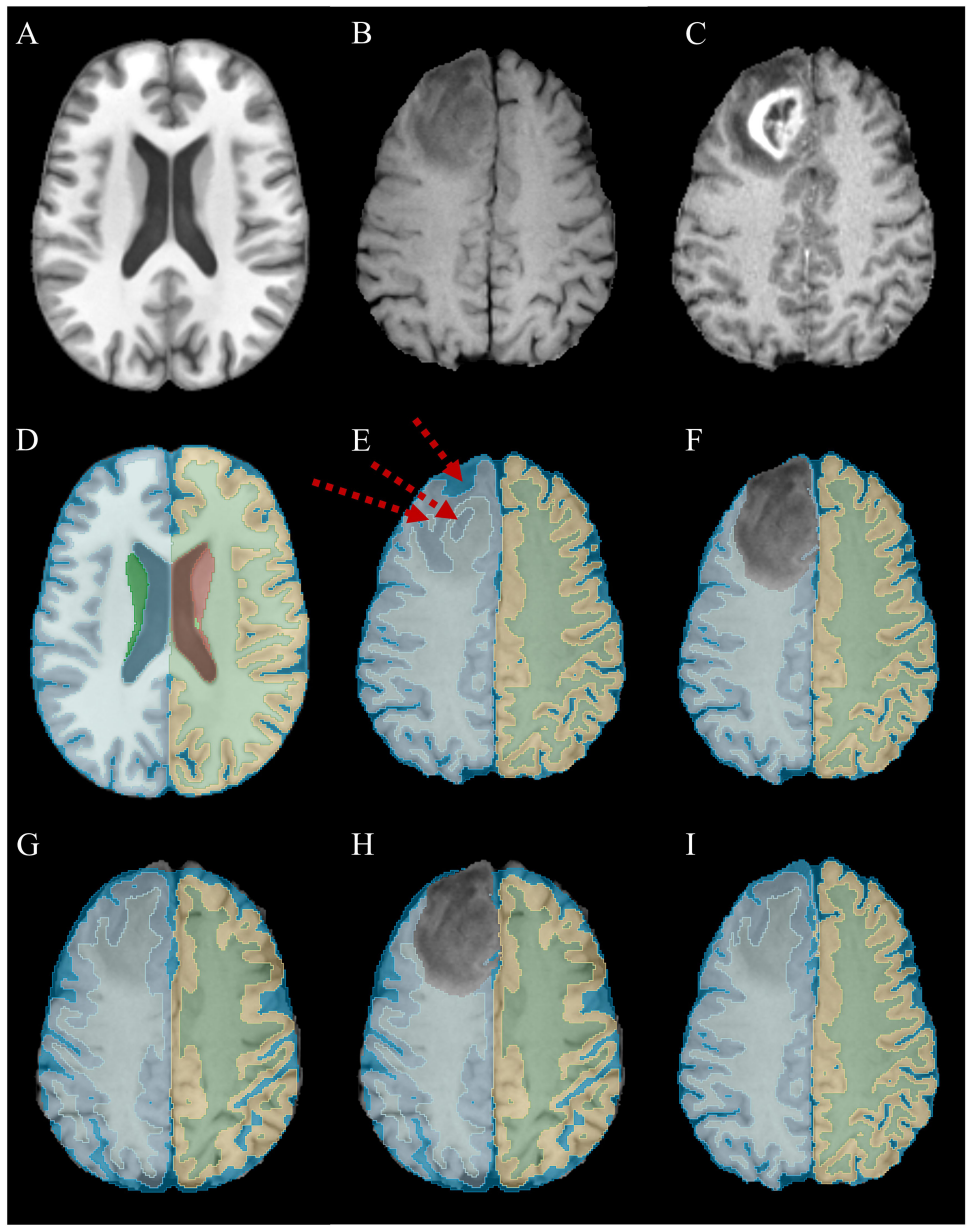
A step-by-step illustration of brain tissue segmentation in the presence of glioma. **(A)** central axial slice of SRI24 atlas in T1 sequence; **(B)** an example of glioma appeared in T1 sequence; **(C)** corresponding T1CE sequence of the same subject depicts tumoral subregions with better contrast, note that all the analyses were done on T1 images; **(D)** the result of parcellated atlas with SynthSeg model (atlas_Synth_); **(E)** the result of SynthSeg model on tumoral data (mask_Synth_) which was failed on/around glioma regions, red arrows highlighting over-segmentation of CSF, and wrongly labeled right cerebral cortex and right cerebral white matter regions; **(F)** masking out the glioma regions (mask_Synth-NoTumor_); **(G)** the result of rigidly registering the parcellated atlas into patient space; **(H)** masking out the glioma regions from the partially aligned atlas allows running a deformable registration between atlas_Synth-NoTumor_ and mask_Synth-NoTumor_; **(I)** warping the atlas_Synth_ with the inverse of obtained deformation fields leads to correct labels within the glioma regions (mask_Final_).

In the second step, we adopted an atlas-based registration strategy to fill the masked-out regions with meaningful labels. To this end, we executed the SynthSeg model on the SRI24 atlas and segmented it into 32 sub-anatomies (atlas_Synth_) since all the studied images were already registered to the SRI24 space. Then, we partially aligned the obtained parcellated atlas into patient anatomy by rigidly registering the atlas_Synth_ into mask_Synth_ for each subject. Similar to the first step, the resulting registered atlas was then multiplied by the GTVs to mask out the tumoral regions (atlas_Synth-NoTumor_). A deformable registration step was executed to register the mask_Synth-NoTumor_ into atlas_Synth-NoTumor_ and correct their misalignments. This step ensures that these two masks are maximally aligned, excluding the tumoral regions. Finally, to determine the anatomical regions within the masked-out areas, the inverse of obtained deformation fields was adopted to warp the atlas_Synth_. This process registers the parcellated atlas into patient anatomy despite the presence of gliomas and mass effects. Therefore, the final registered segmentation masks (mask_Final_) incorporate 32 constituent anatomical structures covering healthy anatomical regions as well as those occupied by the gliomas (see **Figure 1**; **Supplementary Figure 1**).

To quantify how gliomas are distributed in the brain and form a quantitative feature map, we measure the volume of each of the 32 sub-anatomies. Then, by determining the overlapping areas between the GTV masks and the computed mask_Final_, we calculated the ratio of the sub-anatomical volumes affected by the gliomas. In other words, the proposed feature map contains 32 values that vary in the range of 0 to 1, representing the ratio of anatomical structures that are affected by gliomas. As a result, with the proposed *spatial distribution pattern*, the MRI scans of each patient are transformed into a feature vector of length 32. The underlying reason to quantify the anatomical topographic distribution of gliomas is the fact that brain tumors appear in different sizes and shapes at different anatomical locations, and such heterogeneity might affect prognosis and treatment options (23, 24). We hypothesized that the proposed *spatial distribution pattern* would depict how the invasion of gliomas into different sub-anatomies is associated with the underlying genetic attributes and prognostic factors.

### 2.4 Radiomics descriptors

We adopted the radiomic features to quantify the intensity, morphology, and textural attributes of the gliomas by extracting the reproducible features according to the image biomarker standardization initiative on the employed MRI sequences (25, 26). The radiomics pool includes 14 shape-based, 18 first-order statistics, and 70 second-order statistics. Given that morphological (shape-based) features are invariant across imaging sequences, these features were exclusively extracted from the T1ce images. This approach yielded a total of 366 3D radiomic descriptors. It should be mentioned that we followed the guidelines for radiomics studies recommended by CLEAR (27).

### 2.5 Model training, evaluation and validation

A standard machine learning (ML) pipeline was developed to predict the desired class labels for each of the three tasks (MGMT, IDH, OS) based on the extracted handcrafted features. This pipeline includes the following steps: training and validating the performance of learning algorithms with repeated cross-validation (CV) resampling technique, feature selection, model ensembling, and cross-data evaluation. Note that, in the rest of this paper, the ‘combined’ feature set refers to the combination of the proposed *Spatial distribution pattern* and radiomics sets.

The first step involves training a learning algorithm on each of the datasets and for each of the tasks independently. In this study, two learning algorithms, including extreme gradient boosting (XGB), and random forest (RF) were examined as they are recognized as robust and powerful classifiers (28–30). The hyperparameters of these models were tuned independently by running a grid search strategy (31). The prediction power of these models was ranked and the parameters of the top-performing models were stored to instantiate the final learning algorithms and further model ensembling. To reduce the risk of overfitting and removing the irrelevant or partially relevant features, forward feature selection (FFS) was embedded in the training procedure of each cross-validation iteration. Subsets of the most prognostic features selected from the training folds were validated on the validation sets. The described procedure was executed by repeating the 5-fold CV resampling strategy 10 times, with different random seeds for data splitting. Accordingly, the reported metrics are averaged over 250 observed values (10repetition × 5folds × 5models). It is worth mentioning that, to rescale the feature values, min-max normalization was applied to the training subsets and inferred into the validation and test sets. Last but not least, to address class imbalance, the SVM-SMOTE technique was integrated into the training phase. This variant was specifically selected for its superior robustness in high-dimensional spaces compared to standard KNN-based interpolation (32). It is worth highlighting that to prevent data leakage, all feature engineering steps including normalization, class balancing, and feature selection were strictly fitted on training fold data. The resulting transformation parameters were then applied directly to the validation folds and the testing dataset without any further optimization.

To further assess the generalization power of the developed models, we conducted cross-data evaluations. In other words, the models that were developed and validated on each dataset internally were, then, tested on other cohorts as external sets, given the availability of the same class labels. It should be noted that the whole pipeline was tested, independently, on the introduced *spatial distribution pattern*, radiomic set, and their combinations.

In all the experiments, the performance of the models was quantified by employing the following metrics: AUROC, accuracy, sensitivity, and specificity. Finally, the pairwise AUROC comparison method proposed by Delong et al. (33) was employed to assess the statistical significance between the observed values of different feature sets. All the implementations were done in relevant python libraries such as numpy, scikit-learn, and ANTsPy.

## 3 Results

In this section, we first present the quantified statistics resulting from the proposed *spatial distribution pattern*. Then, the prediction power of the employed feature set for each task will be presented in terms of CV and cross-dataset analyses.

### 3.1 Statistics of spatial distribution pattern

Applying the proposed *spatial distribution pattern* resulted in 32 quantitative values representing the percentage of volumes of anatomical structures affected by gliomas. **Table 2** shows the average values of these ratios over the studied datasets. In addition, **Supplementary Figures 2a-d** illustrate detailed statistics of how gliomas were distributed in brain anatomies in the examined datasets.

**Table 2:** Average distribution of glioma in the brain across four datasets, considering the ratio of tumor volume to anatomical structures; L and R stand for left and right.

Anatomical structure	Ratio of volumes	Anatomical structure	Ratio of volumes	Anatomical structure	Ratio of volumes	Anatomical structure	Ratio of volumes
L cerebral white matter	0.093 ± 0.111	L putamen	0.145 ± 0.274	CSF	0.005 ± 0.211	R thalamus	0.101 ± 0.210
L cerebral cortex	0.048 ± 0.059	L pallidum	0.120 ± 0.269	L ventral DC	0.038 ± 0.106	R caudate	0.134 ± 0.263
L lateral ventricle	0.124 ± 0.165	3rd ventricle	0.049 ± 0.147	R cerebral white matter	0.100 ± 0.128	R putamen	0.162 ± 0.299
L inferior lateral ventricle	0.136 ± 0.286	4th ventricle	0.004 ± 0.053	R cerebral cortex	0.050 ± 0.067	R pallidum	0.142 ± 0.299
L cerebellum white matter	0.003 ± 0.037	brain-stem	0.005 ± 0.033	R lateral ventricle	0.140 ± 0.192	R hippocampus	0.090 ± 0.220
L cerebellum cortex	0.002 ± 0.025	L hippocampus	0.095 ± 0.227	R inferior lateral ventricle	0.141 ± 0.304	R amygdala	0.098 ± 0.256
L thalamus	0.075 ± 0.172	L amygdala	0.096 ± 0.254	R cerebellum white matter	0.002 ± 0.038	R accumbens area	0.067 ± 0.203
L caudate	0.115 ± 0.249	L accumbens area	0.024 ± 0.019	R cerebellum cortex	0.001 ± 0.025	R ventral DC	0.047 ± 0.131

The proposed feature set measures the relative size of tumors w.r.t the anatomical structures. To better understand how large the tumors are, compared to the size of anatomical structures, **Supplementary Tables 3**, **4** indicate the absolute volumes of tumoral regions as well as measured brain structures in the 4 studied datasets. For instance, the volume of the left cerebellum cortex which is presented with label 8 varies in the range of 0.056 to 0.058 liter in the examined subjects. Then, the reported value in **Table 2** means that 0.2% of the volume of this structure is affected by gliomas.

**Figure 2:**
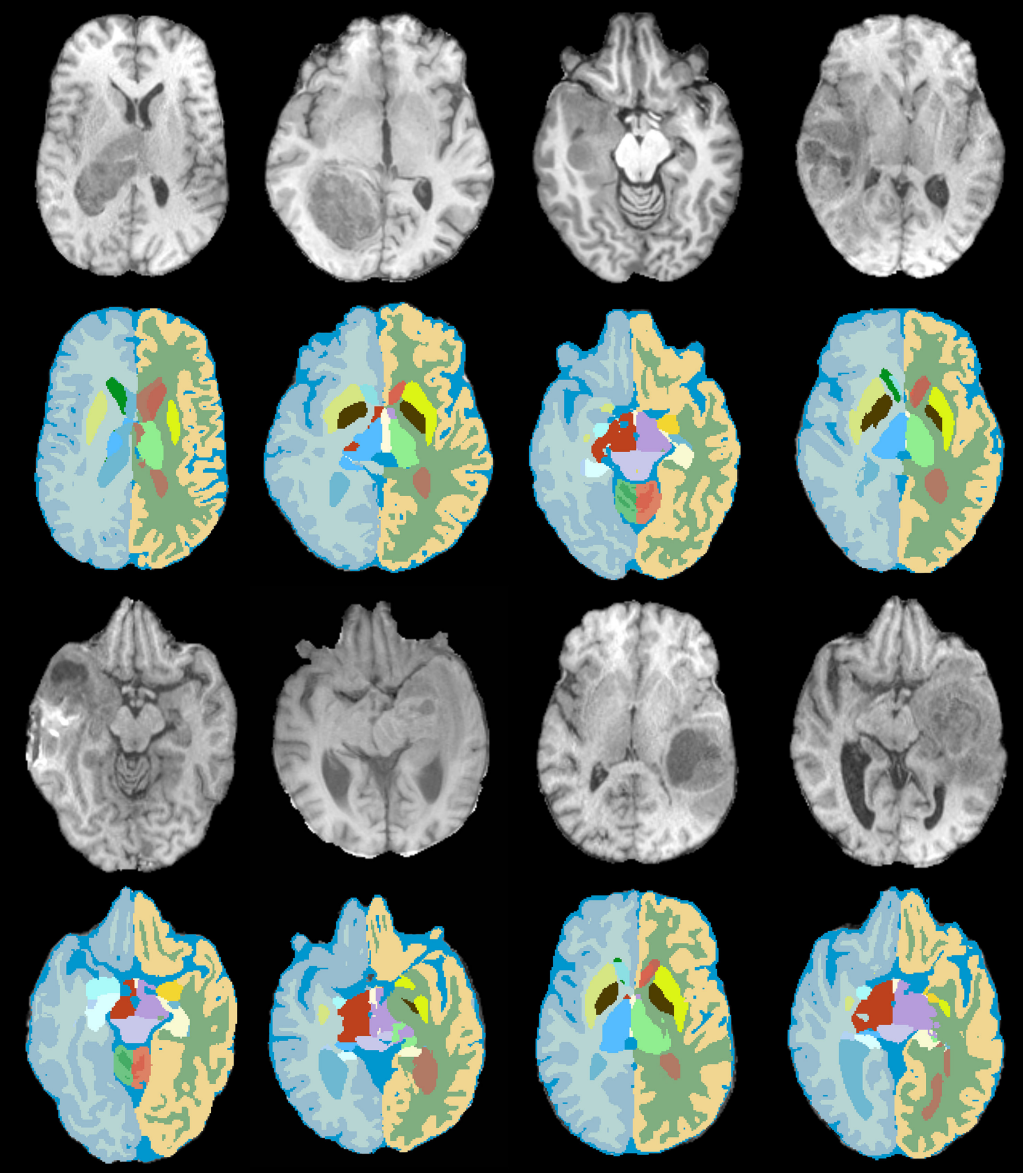
Brain tissue segmentation in the presence of gliomas. An example of 8 challenging cases with large-size tumors that were segmented by the proposed method into 32 constituent anatomical structures.

The largest segmented anatomical structures are left/right cerebral white matter and left/right cerebral cortex. From **Table 2**, the pattern of glioma distribution is rather similar for these structures. In fact, 9.3% of left cerebral white matter, 10% of right cerebral white matter, 4.8% of left cerebral cortex, and 5% of right cerebral cortex were affected by the presence of tumors. **Figure 2** depicts the results of the proposed method on some challenging cases in which the large-size gliomas significantly changed the appearance of normal anatomies.

### 3.2 Results of CV analysis

Extensive ablation studies were conducted to determine the optimal parameters of the employed learning algorithms. To this end, we first mixed all the datasets, then, randomly picked out 20 percent of the data to apply the grid search experiments. **Supplementary Table 2** demonstrates the examined hyperparameters and their investigated range of values for model tuning. For each task, the performance of the algorithms, in terms of AUROC, was sorted and the top 5 performing models were selected for final training in which the full datasets were employed, independently, for CV analysis.

Experimental analysis determined that the most predictive subsets were comprised of 5 features from the *spatial distribution pattern*, 10 from radiomics, and 10 from the combined feature sets. Extending the feature selection beyond these thresholds did not enhance predictive performance, but rather increased model complexity without providing benefits. **Supplementary Figure 4**, **Supplementary Table 5** depict the names and contributions of the selected features to the prediction power for each task. **Tables 3**–**5** represent the performance of CV analysis over the validation sets.

**Table 3:** The results of repeated CV analysis over ensembled models for MGMT methylation status prediction.

MGMT Methylation status – CV analysis					
Dataset	Feature type	Metrics ( μ±σ)			
		AUROC	Accuracy	Sensitivity	Specificity
MICCAI-RSNA	Radiomics	0.643 ± 0.03	0.601 ± 0.04	0.612 ± 0.06	0.597 ± 0.08
	Spatial	0.597 ± 0.07	0.573 ± 0.06	0.588 ± 0.09	0.566 ± 0.05
	Combined	0.645 ± 0.04	0.602 ± 0.04	0.623 ± 0.10	0.580 ± 0.05
UPenn	Radiomics	0.611 ± 0.06	0.608 ± 0.06	0.561 ± 0.12	0.634 ± 0.05
	Spatial	0.577 ± 0.08	0.585 ± 0.07	0.517 ± 0.12	0.651 ± 0.06
	Combined	0.622 ± 0.05	0.630 ± 0.06	0.588 ± 0.08	0.640 ± 0.12
UCSF	Radiomics	0.680 ± 0.06	0.647 ± 0.02	0.637 ± 0.02	**0.682** ± **0.02**
	Spatial	0.631 ± 0.03	0.611 ± 0.04	0.609 ± 0.05	0.669 ± 0.07
	Combined	**0.685** ± **0.08**	**0.655** ± **0.04**	**0.643** ± **0.04**	0.680 ± 0.05
LUMIERE	Radiomics	0.644 ± 0.08	0.597 ± 0.10	0.588 ± 0.37	0.639 ± 0.15
	Spatial	0.608 ± 0.10	0.560 ± 0.06	0.546 ± 0.21	0.615 ± 0.14
	Combined	0.667 ± 0.03	0.617 ± 0.10	0.587 ± 0.36	0.654 ± 0.21

The highest values for each metric are marked as bold.

**Table 4:** The results of repeated CV analysis over ensembled models for IDH status prediction.

IDH Status – CV analysis					
Dataset	Feature type	Metrics ( μ±σ)			
		AUROC	Accuracy	Sensitivity	Specificity
UPenn	Radiomics	0.781 ± 0.04	0.754 ± 0.07	0.731 ± 0.06	0.853 ± 0.02
	Spatial	0.727 ± 0.04	0.706 ± 0.08	0.666 ± 0.02	0.752 ± 0.01
	Combined	0.789 ± 0.07	0.764 ± 0.07	0.729 ± 0.03	0.869 ± 0.01
UCSF	Radiomics	0.961 ± 0.02	0.926 ± 0.02	0.768 ± 0.13	0.976 ± 0.01
	Spatial	0.789 ± 0.02	0.790 ± 0.06	0.678 ± 0.06	0.829 ± 0.03
	Combined	**0.972** ± **0.02**	**0.933** ± **0.03**	**0.816** ± **0.10**	**0.983** ± **0.02**
LUMIERE	Radiomics	0.726 ± 0.13	0.707 ± 0.03	0.657 ± 0.03	0.885 ± 0.03
	Spatial	0.696 ± 0.06	0.665 ± 0.05	0.632 ± 0.10	0.754 ± 0.09
	Combined	0.758 ± 0.11	0.721 ± 0.05	0.681 ± 0.02	0.874 ± 0.02

The highest values for each metric are marked as bold.

**Table 5:** The results of repeated CV analysis over ensembled models for OS time prediction.

OS Time – CV analysis					
Dataset	Feature type	Metrics ( μ±σ)			
		AUROC	Accuracy	Sensitivity	Specificity
UPenn	Radiomics	0.722 ± 0.04	0.691 ± 0.05	**0.758** ± **0.08**	0.688 ± 0.14
	Spatial	0.669 ± 0.05	0.636 ± 0.06	0.629 ± 0.10	0.703 ± 0.09
	Combined	0.736 ± 0.06	0.713 ± 0.03	0.757 ± 0.09	0.702 ± 0.11
UCSF	Radiomics	0.712 ± 0.05	0.702 ± 0.05	0.690 ± 0.09	0.730 ± 0.05
	Spatial	0.675 ± 0.05	0.648 ± 0.04	0.628 ± 0.08	0.679 ± 0.06
	Combined	0.729 ± 0.04	0.716 ± 0.02	0.708 ± 0.09	0.739 ± 0.02
LUMIERE	Radiomics	0.739 ± 0.14	0.719 ± 0.07	0.693 ± 0.23	0.746 ± 0.08
	Spatial	0.680 ± 0.10	0.667 ± 0.08	0.642 ± 0.21	0.712 ± 0.13
	Combined	**0.748** ± **0.11**	**0.736** ± **0.08**	0.713 ± 0.19	**0.762** ± **0.15**

The highest values for each metric are marked as bold.

The reported metrics indicate that the radiomics feature set carries more predictive power than the proposed *spatial distribution pattern*; however, combining these two feature sets yields the highest prediction powers for the conducted experiments. On average, the combined feature sets, in terms of AUROC, improved the prediction power of radiomics by 0.8% in MGMT analysis, 1.7% in IDH analysis, and 1.3% in OS time analysis.

For the MGMT status prediction, the highest prediction power was achieved from the UCSF dataset with an AUROC value of 0.685. This metric remains relatively within the same range (0.622 to 0.667) for the other three datasets. However, one can observe the presence of large variations in the prediction scores of IDH status. In particular, while the UCSF dataset carries the highest prediction power with a value of 0.972, the other two datasets resulted in AUROC values of 0.758 and 0.789. Nevertheless, OS time analysis resulted in rather similar results in which the highest prognostic power comes from the LUMIERE dataset with an AUROC score of 0.748. **Supplementary Table 6** demonstrates the results of the statistical comparison between the examined feature sets.

### 3.3 Results on cross-dataset evaluation

To evaluate the generalization power of the models on external datasets, the best-performing models from CV analyses were selected to be used in the inference mode for predicting the class labels of other datasets. Therefore, the model developed on the UCSF dataset for the MGMT task was applied to the UPenn and LUMIERE datasets. However, since the UCSF dataset is a constituent of the MICCAI-RSNA dataset, this cross-data evaluation was skipped. Likewise, for the IDH task, the UCSF model was applied to the UPenn and LUMIERE datasets as well. Regarding the OS task, the LUMIERE dataset with only 86 subjects outweighed the other sets in CV analyses. For this task, the UPenn dataset resulted in a close performance (0.748 vs. 0.736), nevertheless, it comprises 366 more subjects than LUMIERE. To minimize the risk of overfitting, we picked the UPenn model to be applied to other cohorts. **Tables 6**–**8** show the results of cross-data evaluations. Furthermore, **Supplementary Figures 3** and **4** illustrate the ROC curves, calibration plots, and SHapley Additive exPlanations (SHAP) interpretability analyses for the best-performing models.

**Table 6:** Prediction performance of the MGMT methylation status measured over testing datasets.

MGMT Methylation status – Cross-data testing						
Train data	Test data	Feature type	Metric			
			AUROC	Accuracy	Sensitivity	Specificity
UCSF	UPenn	Radiomics	0.619 ± 0.02	**0.601** ± **0.05**	0.584 ± 0.12	**0.655** ± **0.13**
		Spatial	0.601 ± 0.01	0.572 ± 0.02	0.558 ± 0.03	0.601 ± 0.03
		Combined	**0.628** ± **0.01**	0.601 ± 0.08	0.585 ± 0.19	0.651 ± 0.20
	LUMIERE	Radiomics	0.616 ± 0.03	0.557 ± 0.02	0.576 ± 0.21	0.549 ± 0.09
		Spatial	0.592 ± 0.03	0.540 ± 0.02	0.531 ± 0.08	0.555 ± 0.07
		Combined	0.624 ± 0.03	0.570 ± 0.02	**0.596** ± **0.16**	0.535 ± 0.05

The highest values for each metric are marked as bold.

**Table 7:** Prediction performance of the IDH status measured over testing datasets.

IDH Status – Cross-data testing						
Train data	Test data	Feature type	Metric			
			AUROC	Accuracy	Sensitivity	Specificity
UCSF	UPENN	Radiomics	0.752 ± 0.02	0.737 ± 0.01	**0.714** ± **0.00**	0.802 ± 0.02
		Spatial	0.718 ± 0.01	0.689 ± 0.00	0.636 ± 0.01	0.715 ± 0.01
		Combined	**0.764** ± **0.01**	**0.743** ± **0.00**	0.702 ± 0.00	**0.830** ± **0.01**
	LUMIERE	Radiomics	0.705 ± 0.03	0.691 ± 0.07	0.633 ± 0.06	0.808 ± 0.09
		Spatial	0.675 ± 0.03	0.651 ± 0.01	0.621 ± 0.05	0.717 ± 0.01
		Combined	0.726 ± 0.02	0.709 ± 0.02	0.657 ± 0.04	0.806 ± 0.03

The highest values for each metric are marked as bold.

**Table 8:** Prediction performance of the OS time status measured over testing datasets.

OS time – cross-data testing						
Train data	Test data	Feature type	Metric			
			AUROC	Accuracy	Sensitivity	Specificity
UPENN	UCSF	Radiomics	0.703 ± 0.02	0.701 ± 0.00	0.693 ± 0.02	0.708 ± 0.03
		Spatial	0.652 ± 0.01	0.625 ± 0.01	0.615 ± 0.04	0.656 ± 0.02
		Combined	0.709 ± 0.01	**0.708** ± **0.00**	**0.701** ± **0.01**	0.719 ± 0.02
	LUMIERE	Radiomics	0.695 ± 0.02	0.683 ± 0.00	0.671 ± 0.02	0.714 ± 0.01
		Spatial	0.663 ± 0.02	0.633 ± 0.02	0.612 ± 0.03	0.694 ± 0.04
		Combined	**0.719** ± **0.04**	0.697 ± 0.02	0.674 ± 0.01	**0.742** ± **0.06**

The highest values for each metric are marked as bold.

Similar to CV analyses, radiomic feature set outperformed the proposed *spatial distribution pattern* with rather remarkable margins. Nonetheless, the combined feature sets resulted in the highest prediction scores. The combined feature sets consistently outperformed the radiomic sets. Specifically, the AUROC scores improved by 0.85% for MGMT, 1.65% for IDH, 1.50% for OS. However, generalization remains a challenge. When applying the models to external datasets, performance dropped across all tasks: MGMT: -4.3%, IDH: -3.2%, and OS: -2.9%.

### 3.4 Results summary

In this study, we conducted a comprehensive radiomics analysis for the radiogenomic characterization and survival prediction of gliomas. Additionally, we proposed a novel feature set designed to quantify the spatial distribution of tumors within the brain anatomy and assessed its predictive value when integrated with standard radiomic features. Three distinct clinical tasks include MGMT promoter methylation, IDH mutation status, and OS time prediction, were investigated using three feature strategies (Radiomics, Spatial, and Combined). Detailed experiments were conducted to evaluate model performance under both internal CV and external cross-dataset evaluation settings. To facilitate a comparative analysis of these findings, **Table 9** provides a consolidated summary of the discrimination powers in terms of AUROC achieved by the best-performing models across all experiments and evaluation protocols.

**Table 9:** Summary of the best prediction performance achieved for each clinical task across CV and Cross-Dataset evaluation settings.

Clinical task	CV AUROC (Feature Type)	Dataset CV	Cross-dataset AUROC (Feature Type)	Training => Testing configuration
MGMT	0.685 (Combined)	UCSF	0.628 (Combined)	UCSF=>UPENN
IDH	0.972 (Combined)	UCSF	0.764 (Combined)	UCSF=>UPENN
OS time	0.748 (Combined)	LUMIERE	0.719 (Combined)	UPENN=>LUMIERE

## 4 Discussion

While diagnosis and treatment of brain tumors mainly depend on the pathologic findings, the remarkable degree of inter/intra-tumor heterogeneity of gliomas significantly impacts the intervention options and even the clinical outcomes. Pathology-based evaluation is an inherently localized procedure and, therefore, may not be capable of fully capturing the heterogeneities in genetic patterns of gliomas. On the other hand, preoperative MRIs can provide quantitative global information that might be used for the prediction of glioma subtype and, therefore, influence the choice of treatment options. In recent years, large numbers of studies have been conducted, showing promising results for glioma stratification and prognostic prediction by using MRI data (4). In this work, we assessed the capabilities of radiomics and ML models for predicting the molecular subtypes and treatment outcomes of glioma patients. Additionally, we introduced a novel approach that can quantify the neuroanatomical location of gliomas. We thoroughly evaluated the accuracy and reliability of the radiomics and proposed feature sets for the prediction of MGMT methylation status, IDH status, and OS status.

### 4.1 Spatial distribution pattern

Segmenting the brain anatomy into 32 constituent structures in the presence of brain tumors with the proposed method provides a feature set that quantitatively presents how gliomas are distributed in the brain. Our results show a rather irregular pattern in the distribution of gliomas which were scattered across various brain locations. These findings align with previous research (23, 24). For instance, cerebral white and gray matters in the left and right hemispheres were equally affected by the gliomas. In addition, the right putamen, a small structure involved in movement control, has the highest tumor concentration, with over 16% of its volume affected. Conversely, the right cerebellum cortex, responsible for coordination, shows the least impact, with only 0.1% affected. However, the interpretation needs to consider the relative size of these structures. Large regions like the cerebral cortex (around 0.27 liters) can accommodate tumors with a smaller relative impact compared to tiny structures like the right accumbens area (0.001 liters), where even a small tumor can significantly affect a large portion of the region. Accordingly, brain tumors, in terms of relative size, can fully affect tiny regions while causing less impact on large structures. One surprising finding is that 0.5% of the cerebrospinal fluid (CSF), was affected by the tumor. While gliomas typically do not develop in CSF due to the lack of glial cells, it should be noted that we defined the tumoral regions as a combination of enhancing part, necrosis/non-enhancing as well as peritumoral edema. The edematous regions can contain infiltrative tumor cells and these cells can be presented in the CSF regions. Hence, the impact of tumoral regions on the CSF structures can signify the presence of infiltrative cells.

Employing the *spatial distribution pattern* for predicting the MGMT methylation, IDH mutation, and OS time status resulted in inferior performance compared to radiomic features in all the experiments. In fact, the proposed feature set aims at quantifying only the spatial distribution of the gliomas and cannot be directly compared to radiomics which are designed to capture intensity, morphological, and textural attributes. However, integrating the proposed feature set into radiomic pools consistently improved the discrimination power in different experiments. Indeed, the proposed spatial distribution pattern can provide global information regarding the impacts of gliomas on brain anatomies in contrast to radiomics which only quantifies the localized characteristics of tumoral regions. Therefore, the proposed method can be considered as a complementary feature set to radiomic pools. The selected features from the spatial distribution pattern were different for the three studied tasks. This observation implies that the impacted anatomical structures are not equally correlated with each of the MGMT methylation, IDH mutation, and OS time status. Additionally, in the combined feature set, textural radiomics and *spatial distribution patterns* frequently contributed to the build of most prognostic feature subsets when the FFS method was applied. The proposed feature set presents a valuable opportunity for quantifying the spatial distribution of infiltrative glioma cells. Recent findings indicate that the distribution of these cells, particularly those extending beyond the visible margins of the GTV, is correlated with OS outcomes (34, 35). Accordingly, the described *spatial distribution pattern* can be leveraged to estimate the precise anatomical spread of infiltrative cells within the brain.

Beyond improved prediction, the proposed method offers several advantages. It relies on simple, size-based measurements, making the results easily interpretable by clinicians. Additionally, the segmentation technique used to analyze brain structures can be applied to other brain tumor studies, such as investigating recurrence patterns and tracking changes in brain structures over time.

### 4.2 MGMT methylation status prediction

Our analysis of 1398 subjects across 4 datasets revealed a concerning limitation in predicting MGMT status. The highest achieved prediction power in terms of AUROC was only 0.685. This performance remained consistently poor across datasets and even dropped by 5.7% when the best-trained model was applied to other datasets during the cross-data testing phase, which resulted in the highest AUROC score of 0.628. To technically evaluate such poor results, it should be emphasized that the applied grid searches for tuning the model hyperparameters assured us that the developed models were not underfitted. Moreover, integrating the feature selection technique to keep only a few prognostic features, training the models with repeated CV strategy, and further ensembling the top-performing models increased our confidence that the developed models were not overfitted either. In this context, it should be noted that the MGMT labels were almost equally distributed between the binary classes; thus, the models were not biased toward the majority class. In particular, the percentage of methylated and unmethylated MGMTs was 0.52 vs. 0.48 for the RSNA dataset, 0.44 vs. 0.56 for the Upenn dataset, and 0.46 vs. 0.54 for the LUMIERE dataset. We adopted the SMOTE technique to deal with the imbalance distribution in the UCSF dataset (0.72 vs. 0.28). Accordingly, it can be derived that the observed poor prediction powers were not due to methodological issues. In other words, prediction scores of MGMT status remained poor regardless of the studied data, examined feature set, or training strategies. This finding is further substantiated by the calibration curves (**Supplementary Figure 3**). Although the combined feature set demonstrates slight adherence to the ideal diagonal compared to individual feature sets in the mid-probability range, all models exhibit suboptimal performance in this interval. The observed behavior of the curves suggests a limited discriminative capacity, indicating that the classifiers struggle to reliably stratify moderate-risk and high-risk cases.

Our observations are in agreement with the findings of recent studies investigating the potential correlations between MRI data and MGMT status.

As detailed in **Supplementary Table 7**, our results (AUROC 0.645) are competitive with recent literature using the RSNA dataset. While some studies on small-scale or private datasets report higher accuracy (41, 42), studies using the public RSNA benchmark challenge (39) consistently report AUROCs below 0.65 (36–38, 40). While these discrepancies in the observed performance can be caused by a variety of reasons such as the inclusion of lower-grade tumors in the examined cohorts, and different techniques for determining the MGMT status in the laboratory, lack of external validation steps, and over-reliance on certain variables could be other important factors. Accordingly, it is strongly recommended to develop reproducible models on heterogeneous datasets and further evaluate their performance on external testing sets.

Our comprehensive experiments yielded poor prediction power, suggesting that current imaging biomarker models, particularly those utilizing structural MRIs, are inadequate for capturing meaningful correlations between imaging data and MGMT methylation status. Consequently, future research should explore alternative MRI sequences, such as perfusion and diffusion-weighted imaging, instead of using more advanced ML/DL models. More importantly, integrating imaging biomarkers with genetic and clinical data for multi-omics analyses holds promise for improved prediction.

### 4.3 IDH status prediction

In contrast to MGMT analysis, promising results were achieved for the prediction of IDH mutation status, demonstrating the potential of imaging data as a non-invasive measurement for tumor stratification purposes. After analyzing 1180 subjects from 3 different datasets, the most accurate model was developed over the UCSF dataset, which resulted in an average AUROC value of 0.972. It should be noted that the UCSF dataset contains a relatively good balance between the wildtype group with 398 subjects and the mutant group with 103 subjects. On the other hand, the distributions of class labels in the Upenn and LUMIERE cohorts were strongly skewed toward the wildtype IDH group. Specifically, 98% of subjects in the Upenn dataset and 96% of the LUMIERE cohort belonged to the wildtype class. Such severe imbalanced distributions cannot be compensated with resampling or augmentation techniques. Therefore, we reformulated the binary classification problem for these two cohorts as wildtype IDH class against the rest of the classes, which includes mutant, negatives, and NOS/NEC. Accordingly, the percentage of wildtype IDHs against the other class became 0.81 vs. 0.19 for the Upenn dataset and 0.83 vs. 0.17 for the LUMIERE cohort. While this label reformulation slightly improved the imbalanced issues, it essentially led to presenting multiple subclasses in one single group. Therefore, the highest prediction power over the Upenn and LUMIERE datasets (0.758 to 0.789) is not comparable to those of the UCSF cohort. We believe that the lower yet promising prediction powers achieved for these two cohorts can be justified by (i) highly imbalanced distributions of class labels, and (ii) classification complexity caused by label reformulation. Subsequently, applying the UCSF model to the Upenn and LUMIERE datasets in the cross-data testing step resulted in a significant reduction of the classification power of the UCSF dataset alone. Nonetheless, the AUROC metrics in the test phase were in the range of 0.726 to 0.764, which is relatively similar to the range of their CV analysis step. Additionally, no significant changes in the quantified values of the accuracy, sensitivity, and specificity metrics imply that the learned models are not biased toward either of the binarized classes.

Our proposed pipeline yields competitive classification power compared to other studies that developed and tested their models on the UCSF dataset. In this domain, the reported AUROC metrics on CV analysis with a variety of radiomics and end-to-end DL models lie in the range of 0.87 to 0.964 (30, 36, 43). Besides the UCSF dataset, the reported metrics from studies that predicted IDH status by running cross-data analysis show robustness in model performance with only limited drops of accuracy when tested on external datasets, resulting in the classification power of 0.84 to 0.86 (44–46).

The IDH status prediction task yields the most stable calibration curves. While the spatial features alone are prone to error in high-probability regions, the radiomics and combined sets closely approximate the ideal diagonal, indicating trustworthy predictive capacity. Despite a slight systemic over-confidence, these models show superior alignment with reality compared to the other tasks. With the promising results we achieved for IDH mutation status and the lack of inconsistency in the reported results by other studies, it can be inferred that structural MRI data incorporate quantitative characteristics of glioma that are correlated with IDH mutation state. Nevertheless, relying on the models that were tested on limited datasets could be misleading; therefore, the reliability of imaging biomarker models for IDH status prediction should be further investigated by testing these models on multiple large-scale and heterogeneous datasets.

### 4.4 Overall survival prediction

Our experiments for predicting the OS time in terms of a binary status (short-term vs. long-term survivors) over a total number of 1308 subjects from 3 datasets resulted in relatively stable models. The highest discrimination scores for the CV analysis lie in the range of 0.729 to 0.748. Similar to the MGMT and IDH experiments, classification power was dropped when the best model achieved from the CV analysis evaluated on the testing datasets, which resulted in the highest value of 0.719. Different factors, including imaging vendors, acquisition settings, and patient populations, can cause such domain shifts between the training and testing cohorts. Our observations are in agreement with other relevant studies using the same type of image data for OS time predictions. In this domain, the developed radiomics and DL models resulted in robust prediction powers of up to 0.78 and dropped to 0.67 when testing on external datasets (12, 47–49). It is worth mentioning that applying the median survival time as a hard threshold to binarize the OS status addresses the problem of class imbalance issues. Even for this simplified binary survival analysis, calibration curves display significant noise evidenced by oscillatory patterns. These deviations point to potential data sparsity within the Lumiere dataset and more importantly the models’ inability to converge on a stable signal, highlighting the complexity of modeling survival outcomes. While such a binary problem facilitates the development of ML/DL pipelines, in many applications, other types of predictions, including three-class classification and continuous-time prediction for survival analysis, are of interest. Nevertheless, the prediction powers of such scenarios remain limited despite the advances in the field of DL techniques (50, 51). Accordingly, it can be deduced that structural MRI data can provide a relatively meaningful correlation with the binarized OS time status of glioma patients; however, these image data are not sufficient to derive solid models for other survival analysis applications. Hence, more investigations are required to examine the potential of alternative MRI sequences as well as the combination of image data with laboratory and clinical factors for developing more robust and reliable models for survival analysis.

While imaging biomarker models show promise for various glioma-related tasks, their robustness and reliability require extensive validation and reproducibility testing. International benchmarking competitions (52), like those established by the BraTS community, offer an objective benchmarking framework through glioma diagnosis and prognosis prediction challenges. In this study, we compared MGMT methylation and OS time prediction against the reported results by related challenges, while we evaluated the IDH mutation prediction on 3 independent public datasets due to the absence of a dedicated challenge.

This study acknowledges some limitations which can be addressed in future studies. First, the potential bias due to imbalanced structure size in the proposed segmentation method can be addressed by incorporating alternative structural or functional atlases for more uniform segmentation. Second, the restricted use of structural MRI sequences calls for further investigation into the potential performance improvements gained by incorporating perfusion and diffusion MRI sequences. Finally, future studies should explore the impact of preprocessing steps like bias field correction and intensity normalization on the generalizability of the models.

## 5 Conclusion

Our experiments indicate that the imaging signature, derived by combining the proposed feature set with radiomics, exhibits a strong correlation with IDH mutation status (AUROC: CV = 0.972, Cross-data = 0.764). In contrast, we observed a moderate correlation with OS time (AUROC: CV = 0.748, Cross-data = 0.719) and a weak correlation with MGMT methylation status (AUROC: CV = 0.685, Cross-data = 0.628). These findings suggest the promising utility of imaging biomarkers for IDH prediction but highlight limitations in using solely image data for the other two tasks.

## Data Availability

The original contributions presented in the study are included in the article/**Supplementary Material**. Further inquiries can be directed to the corresponding author.

## Footnotes

1 Not otherwise specified (NOS), Not elsewhere classified (NEC).
