# Supplementary material for: A comprehensive evaluation of MRI-based radiogenomics and prognosis prediction in glioma
Source: Front Oncol. 2026 Jan 5;15:1679634. doi: 10.3389/fonc.2025.1679634 (PMC12812525; doi:10.3389/fonc.2025.1679634)
Supplement: Supplementary file 1 [file DataSheet1.docx]

Supplementary Material

A Comprehensive Evaluation of MRI-based Radiogenomics and Prognosis Prediction in Glioma

Mehdi Astaraki^1,2,3*^, Marta Lazzeroni^1.2^, Iuliana Toma-Dasu^1,2^

^1^Division of Medical Radiation Physics, Department of Physics, Stockholm University, Stockholm, Sweden

^2^Department of Oncology-Pathology, Karolinska Institutet, Stockholm, Sweden

^3^Department of something else, Karolinska Institutet, Stockholm, Sweden

Table 1. List of anatomical structures and corresponding class labels. Note that the label values are in line with FreeSurfer^[[1]](#footnote-1)^ classification guidelines. The order of the structures is compatible with the posterior view.

| Structure Name | Class Label | Structure Name | Class Label |
| --- | --- | --- | --- |
| left cerebral white matter | 2 | CSF | 24 |
| left cerebral cortex | 3 | left ventral DC | 28 |
| left lateral ventricle | 4 | right cerebral white matter | 41 |
| left inferior lateral ventricle | 5 | right cerebral cortex | 42 |
| left cerebellum white matter | 7 | right lateral ventricle | 43 |
| left cerebellum cortex | 8 | right inferior lateral ventricle | 44 |
| left thalamus | 10 | right cerebellum white matter | 46 |
| left caudate | 11 | right cerebellum cortex | 47 |
| left putamen | 12 | right thalamus | 49 |
| left pallidum | 13 | right caudate | 50 |
| 3rd ventricle | 14 | right putamen | 51 |
| 4th ventricle | 15 | right pallidum | 52 |
| brain-stem | 16 | right hippocampus | 53 |
| left hippocampus | 17 | right amygdala | 54 |
| left amygdala | 18 | right accumbens area | 58 |
| left accumbens area | 26 | right ventral DC | 60 |


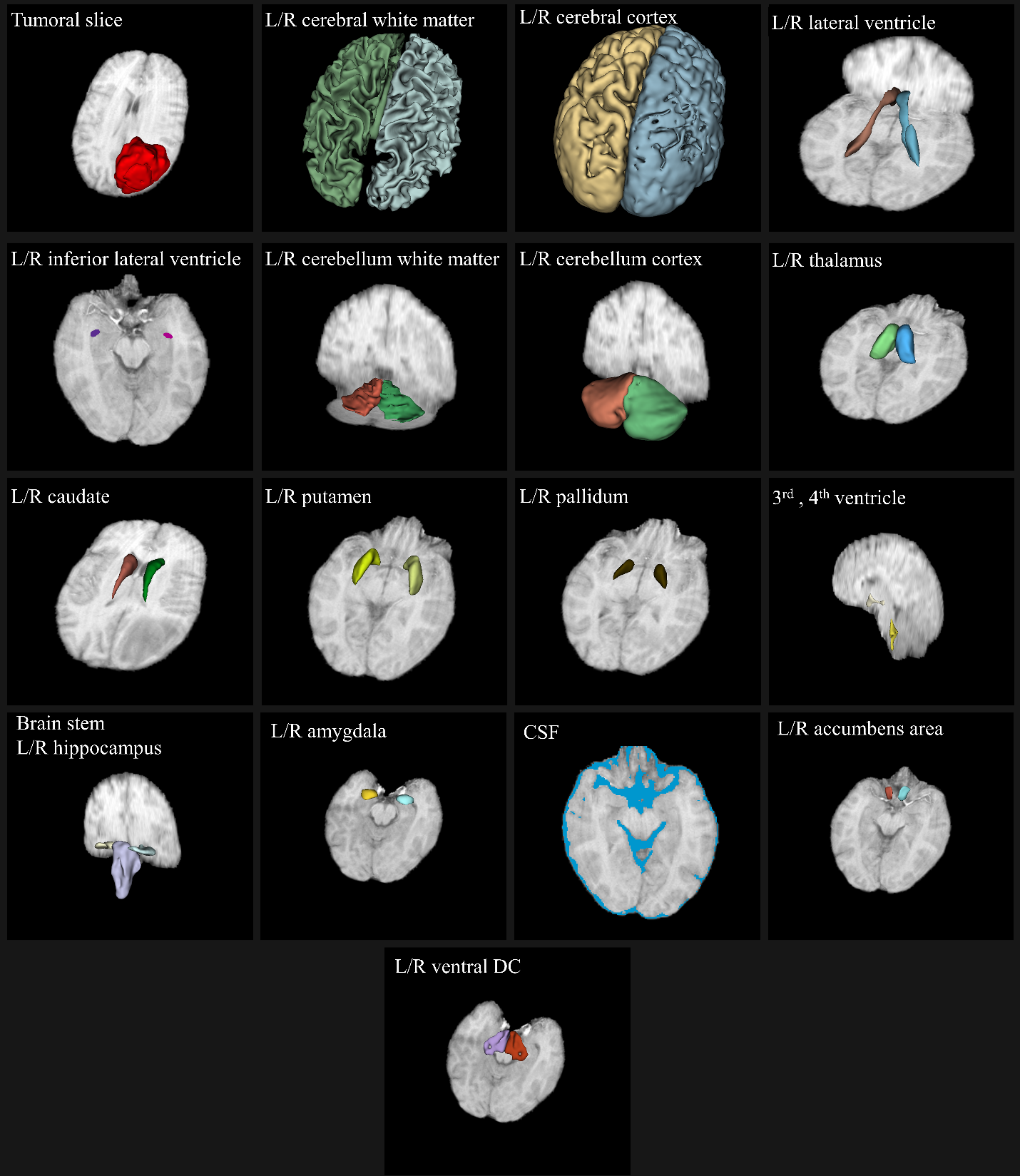


Figure 1. Visualization of the segmented brain structures in a subject with a large-size tumor. Each image is presented in a certain view that best demonstrates the segmented regions. L/R refers to left and right hemispheres.


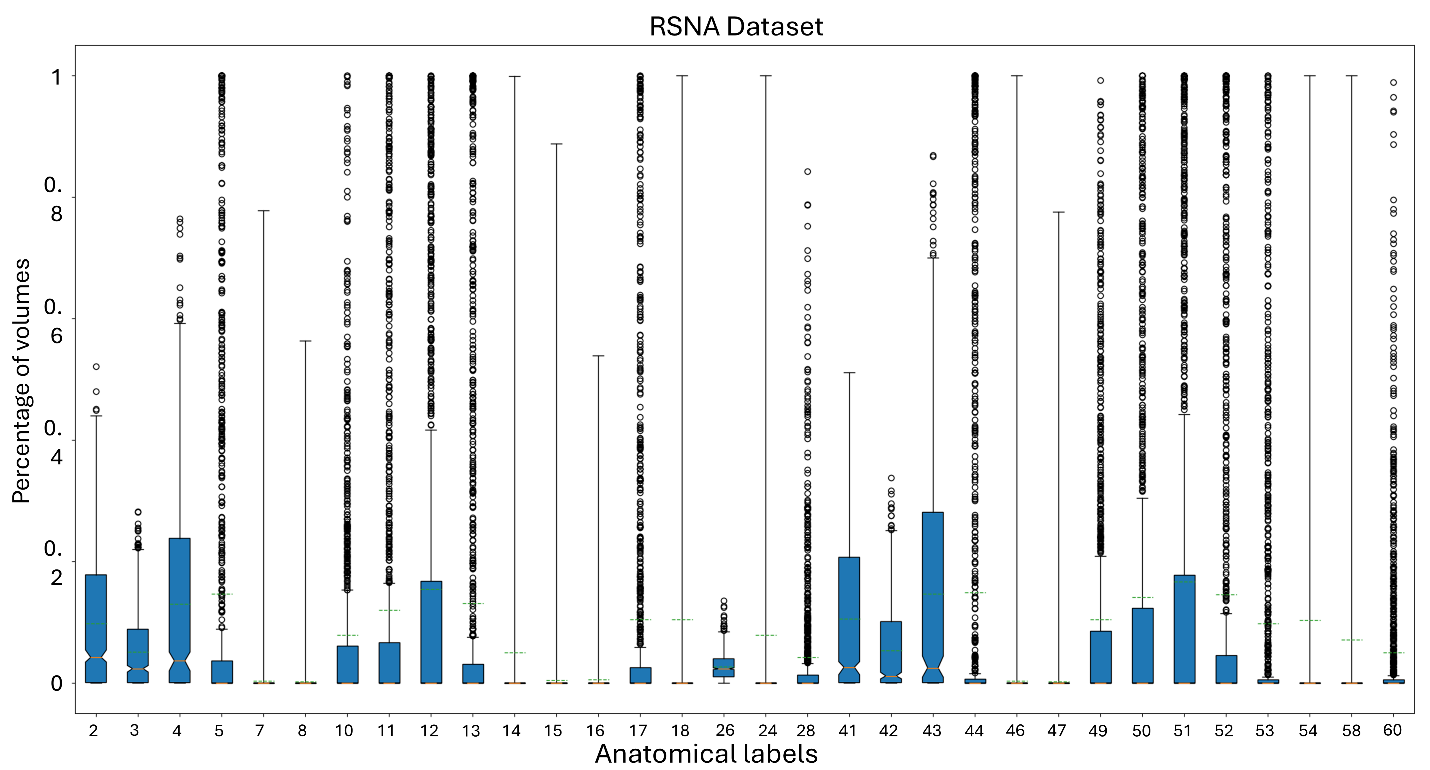


Figure 2a. Boxplot of the distribution of tumors in brain anatomy in terms of percentage of volume for RSNA dataset.


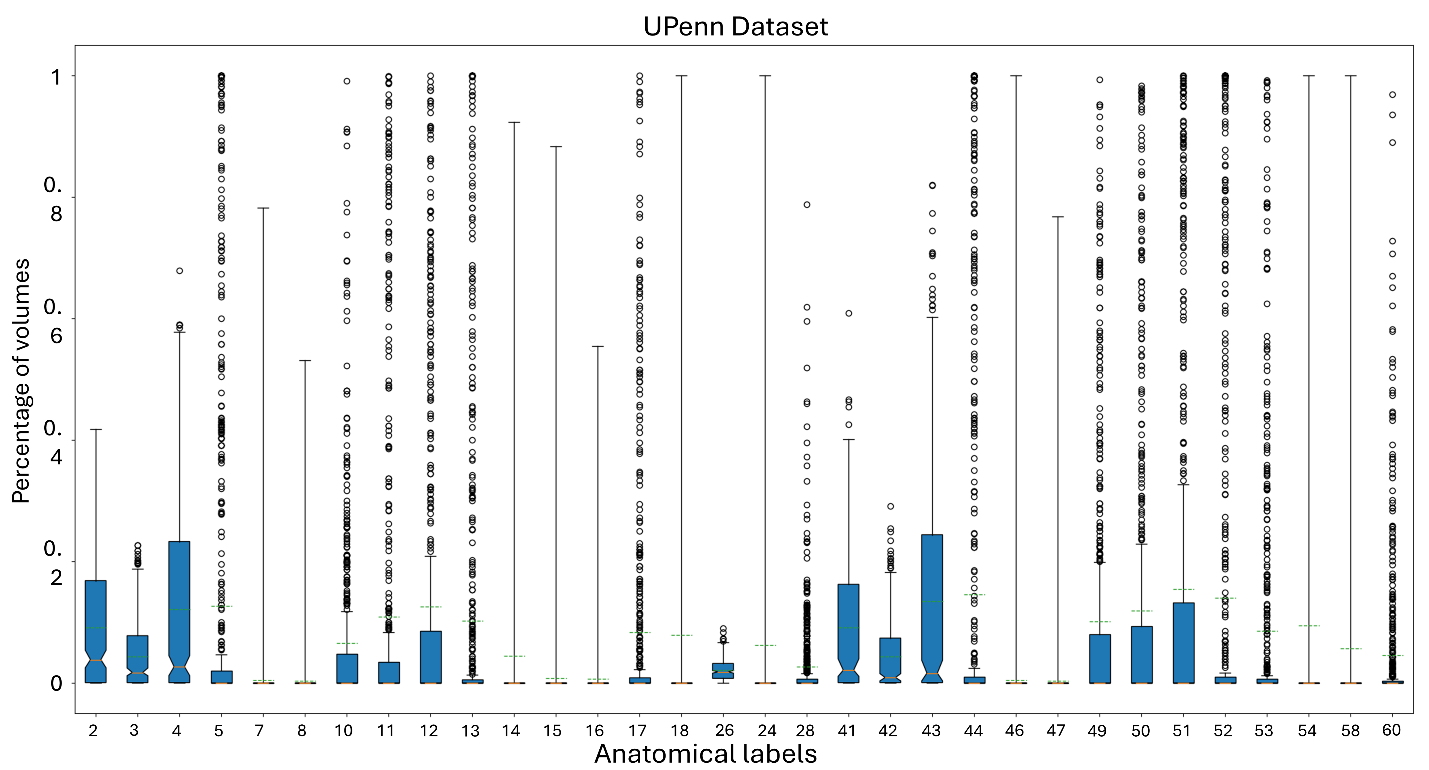


Figure 2b. Boxplot of the distribution of tumors in brain anatomy in terms of percentage of volume for UPenn dataset.


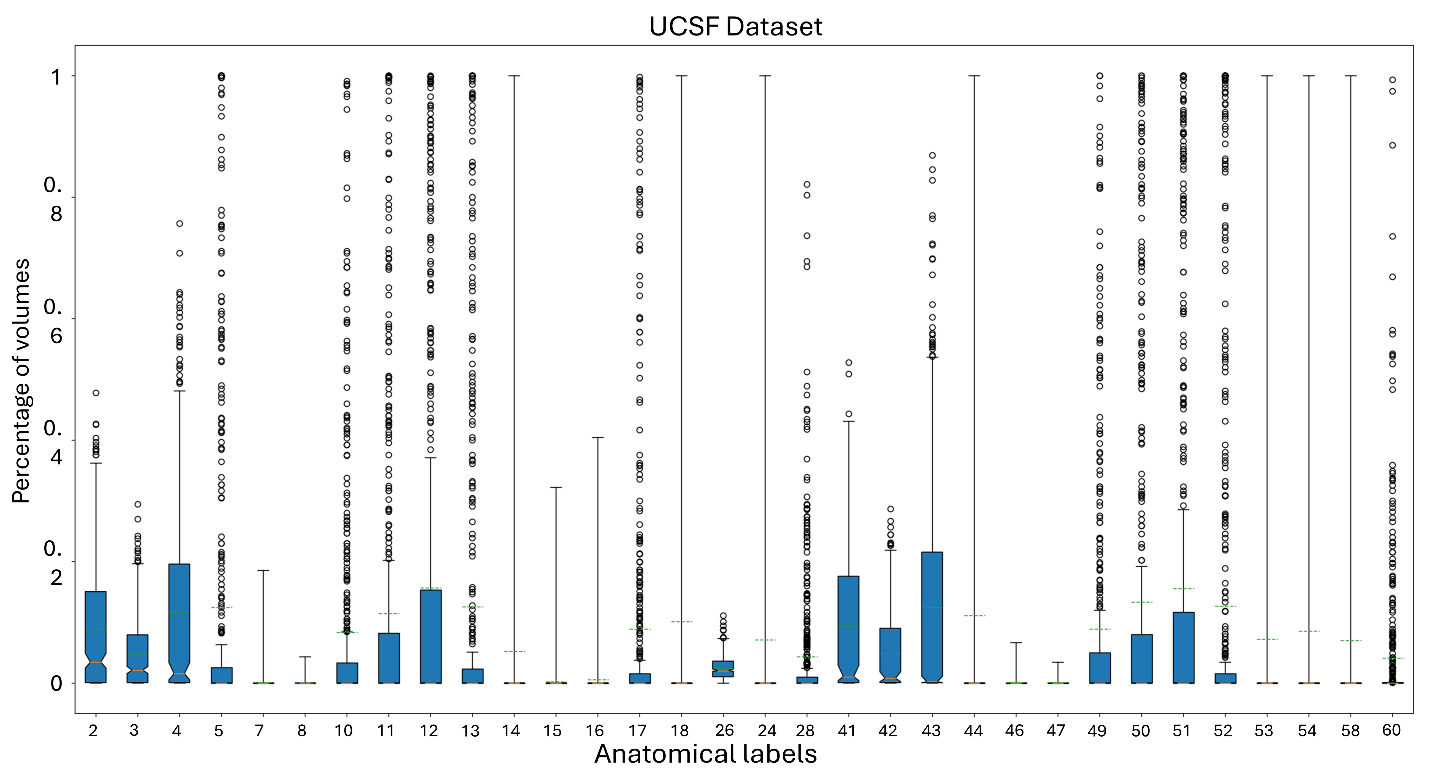


Figure 2c. Boxplot of the distribution of tumors in brain anatomy in terms of percentage of volume for UCSF dataset.


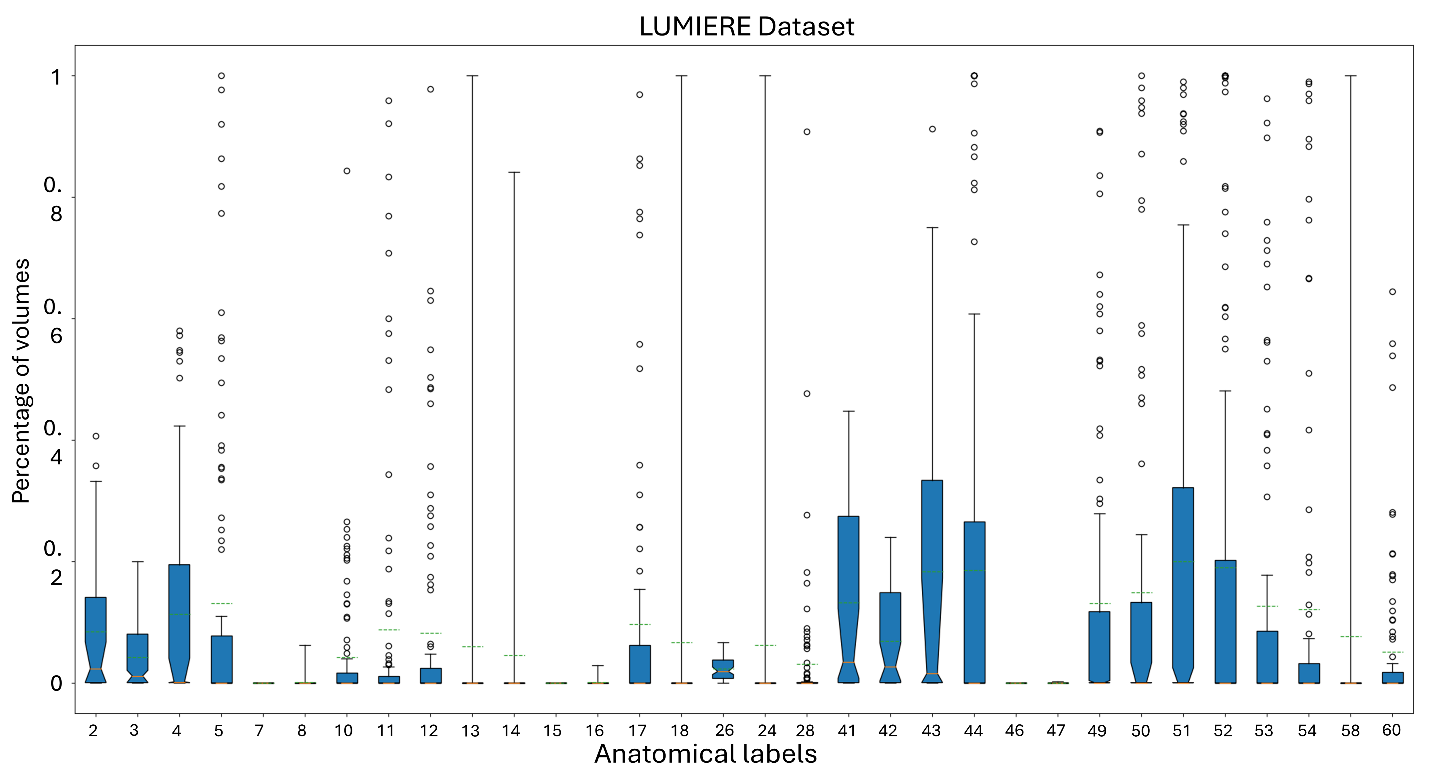


Figure 2d. Boxplot of the distribution of tumors in brain anatomy in terms of percentage of volume for the LUMIERE dataset.


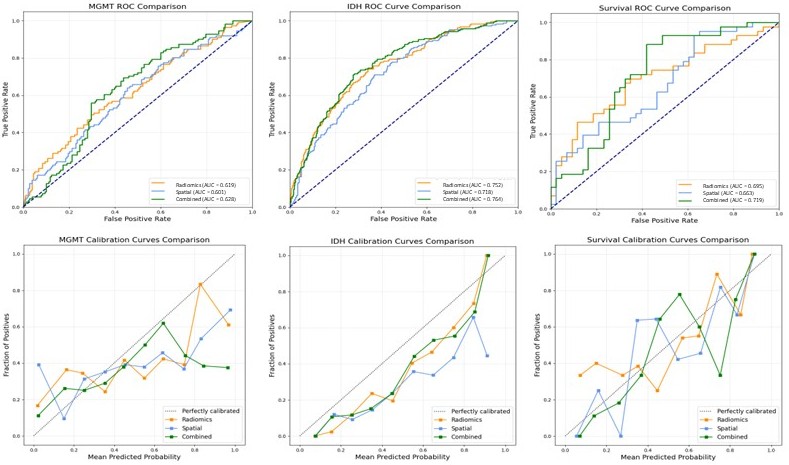


Figure 3. Performance comparison of radiomics (red), spatial (blue), and combined (green) feature sets. The upper row displays ROC curves, while the lower row illustrates calibration curves for the best cross-dataset models. Columns correspond to the specific prediction targets: MGMT status (left), IDH mutation (center), and survival (right).


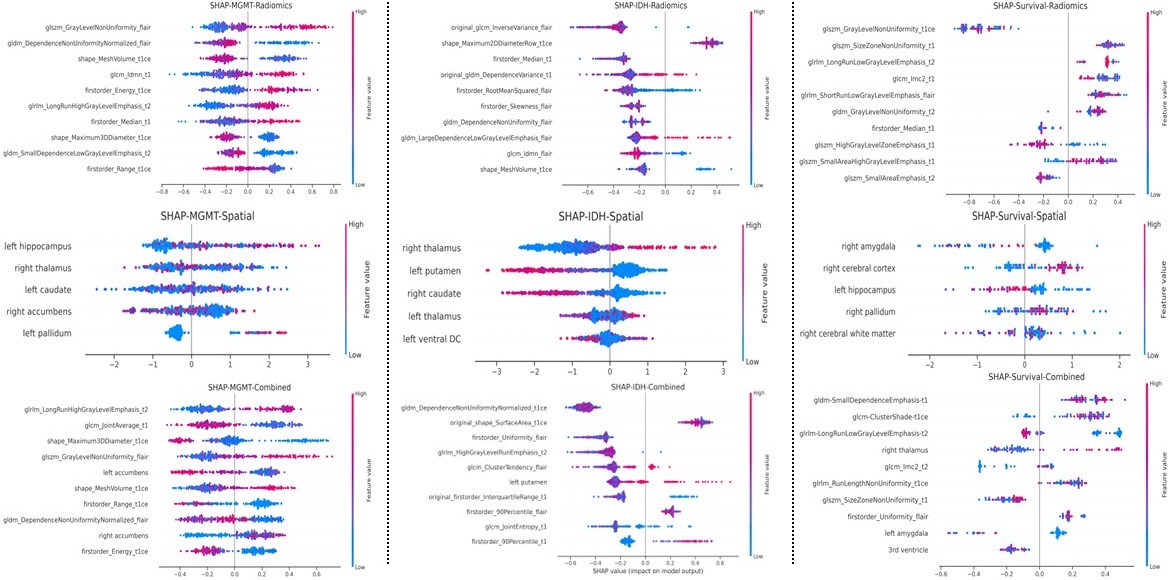


Figure 4. SHAP (SHapley Additive exPlanations) summary plots illustrating feature importance and impact on model output for the best-performing cross-dataset models. Panels are organized by prediction task (columns: MGMT, IDH, and survival) and feature set (rows: radiomics, spatial, and combined).

Table 2. Range of investigated values in grid search experiments for tuning the hyperparameters of the examined learning algorithms.

| Model | Hyperparameter | Range of values |
| --- | --- | --- |
| random forest | n_estimators | [50,80100,200,300,500,600,800,1000] |
| random forest | max_depth | [3,4,5,6,7,8,9,10] |
| random forest | max_leaf_nodes | [4,5,6,7,8, 9,10,12,15,18,20,25,30,50,80,100] |
| extreme gradient boosting | learning_rate | [0.15, 0.2, 0.25, 0.30] |
| extreme gradient boosting | min_child_weight | [0.5, 1, 1.5] |
| extreme gradient boosting | subsample | [0.5, 0.8] |
| extreme gradient boosting | colsample_bytree | [0.2, 0.5, 1] |
| extreme gradient boosting | max_depth | [5, 8, 10, 20, 30] |
| extreme gradient boosting | n_estimators | [50,80,100, 200, 300, 500, 1000, 2000] |

Table 3. The volume of glioma subregions in terms of liter over the studied datasets ($\boldsymbol{\mu}\boldsymbol{\pm}\boldsymbol{\sigma}$)

| Dataset | Enhancing tumor | Necrosis/non-enhancing | Edematous/infiltrated |
| --- | --- | --- | --- |
| RSNA | 0.022$\pm$0.01 | 0.014$\pm$0.02 | 0.060$\pm$0.04 |
| UPenn | 0.018$\pm$0.01 | 0.011$\pm$0.01 | 0.054$\pm$0.03 |
| UCSF | 0.020$\pm$0.01 | 0.012$\pm$0.01 | 0.061$\pm$0.04 |
| LUMIERE | 0.017$\pm$0.01 | 0.016$\pm$0.01 | 0.067$\pm$0.04 |

Table 4. The volume of the segmented anatomical structures in terms of liter over the studied datasets ($\boldsymbol{\mu}\boldsymbol{\pm}\boldsymbol{\sigma}$)

| Anatomical Label | RSNA | UPenn | UCSF | LUMIERE |
| --- | --- | --- | --- | --- |
| 2 | 0.239$\pm$0.03 | 0.241$\pm$0.02 | 0.250$\pm$0.03 | 0.239$\pm$0.02 |
| 3 | 0.271$\pm$0.02 | 0.275$\pm$0.02 | 0.285$\pm$0.02 | 0.278$\pm$0.02 |
| 4 | 0.014$\pm$0.01 | 0.015$\pm$0.01 | 0.014$\pm$0.01 | 0.014$\pm$0.00 |
| 5 | 0.001$\pm$0.00 | 0.001$\pm$0.00 | 0.001$\pm$0.00 | 0.000$\pm$0.00 |
| 7 | 0.015$\pm$0.00 | 0.015$\pm$0.00 | 0.016$\pm$0.00 | 0.015$\pm$0.00 |
| 8 | 0.056$\pm$0.00 | 0.057$\pm$0.00 | 0.058$\pm$0.00 | 0.056$\pm$0.00 |
| 10 | 0.008$\pm$0.00 | 0.008$\pm$0.00 | 0.008$\pm$0.00 | 0.007$\pm$0.00 |
| 11 | 0.004$\pm$0.00 | 0.004$\pm$0.00 | 0.004$\pm$0.00 | 0.004$\pm$0.00 |
| 12 | 0.006$\pm$0.00 | 0.006$\pm$0.00 | 0.006$\pm$0.00 | 0.005$\pm$0.00 |
| 13 | 0.001$\pm$0.00 | 0.001$\pm$0.00 | 0.001$\pm$0.00 | 0.001$\pm$0.00 |
| 14 | 0.001$\pm$0.00 | 0.001$\pm$0.00 | 0.001$\pm$0.00 | 0.001$\pm$0.00 |
| 15 | 0.002$\pm$0.00 | 0.002$\pm$0.00 | 0.002$\pm$0.00 | 0.002$\pm$0.00 |
| 16 | 0.025$\pm$0.00 | 0.024$\pm$0.00 | 0.026$\pm$0.00 | 0.025$\pm$0.00 |
| 17 | 0.004$\pm$0.00 | 0.004$\pm$0.00 | 0.004$\pm$0.00 | 0.004$\pm$0.00 |
| 18 | 0.002$\pm$0.00 | 0.002$\pm$0.00 | 0.002$\pm$0.00 | 0.002$\pm$0.00 |
| 24 | 0.190$\pm$0.03 | 0.243$\pm$0.03 | 0.197$\pm$0.03 | 0.206$\pm$0.02 |
| 26 | 0.001$\pm$0.00 | 0.001$\pm$0.00 | 0.001$\pm$0.00 | 0.001$\pm$0.00 |
| 28 | 0.004$\pm$0.00 | 0.004$\pm$0.00 | 0.005$\pm$0.00 | 0.004$\pm$0.00 |
| 41 | 0.237$\pm$0.02 | 0.237$\pm$0.02 | 0.248$\pm$0.03 | 0.242$\pm$0.02 |
| 42 | 0.272$\pm$0.02 | 0.275$\pm$0.02 | 0.284$\pm$0.02 | 0.279$\pm$0.03 |
| 43 | 0.014$\pm$0.01 | 0.014$\pm$0.01 | 0.013$\pm$0.00 | 0.013$\pm$0.00 |
| 44 | 0.001$\pm$0.00 | 0.001$\pm$0.00 | 0.001$\pm$0.00 | 0.000$\pm$0.00 |
| 46 | 0.015$\pm$0.00 | 0.014$\pm$0.00 | 0.015$\pm$0.00 | 0.015$\pm$0.00 |
| 47 | 0.054$\pm$0.00 | 0.056$\pm$0.00 | 0.057$\pm$0.00 | 0.054$\pm$0.00 |
| 49 | 0.008$\pm$0.00 | 0.008$\pm$0.00 | 0.008$\pm$0.00 | 0.008$\pm$0.00 |
| 50 | 0.004$\pm$0.00 | 0.004$\pm$0.00 | 0.004$\pm$0.00 | 0.004$\pm$0.00 |
| 51 | 0.005$\pm$0.00 | 0.006$\pm$0.00 | 0.006$\pm$0.00 | 0.005$\pm$0.00 |
| 52 | 0.002$\pm$0.00 | 0.002$\pm$0.00 | 0.002$\pm$0.00 | 0.002$\pm$0.00 |
| 53 | 0.004$\pm$0.00 | 0.004$\pm$0.00 | 0.004$\pm$0.00 | 0.004$\pm$0.00 |
| 54 | 0.002$\pm$0.00 | 0.002$\pm$0.00 | 0.002$\pm$0.00 | 0.002$\pm$0.00 |
| 58 | 0.001$\pm$0.00 | 0.001$\pm$0.00 | 0.001$\pm$0.00 | 0.001$\pm$0.00 |
| 60 | 0.004$\pm$0.00 | 0.004$\pm$0.00 | 0.004$\pm$0.00 | 0.004$\pm$0.00 |

Table 5. Name of the selected features for each of the examined feature types. Note that the name of radiomic features is followed by the name of MR sequences from which it was extracted.

| Task | Feature type | Selected Features |
| --- | --- | --- |
| MGMT Methylation Status | Spatial distribution pattern | left caudate, left pallidum, left hippocampus, right thalamus, right accumbens area |
|  | Radiomics | firstorder_Median_t1, glcm_Idmn_t1, shape_Maximum3DDiameter_t1ce, shape_MeshVolume_t1ce, firstorder_Energy_t1ce, firstorder_Range_t1ce, glszm_GrayLevelNonUniformity_flair, gldm_DependenceNonUniformityNormalized_flair, glrlm_LongRunHighGrayLevelEmphasis_t2, gldm_SmallDependenceLowGrayLevelEmphasis_t2 |
|  | Combined | glcm_JointAverage_t1, glcm_Idmn_t1, shape_Maximum3DDiameter_t1ce, shape_MeshVolume_t1ce, firstorder_Energy_t1ce, firstorder_Range_t1ce, glszm_GrayLevelNonUniformity_flair, gldm_DependenceNonUniformityNormalized_flair, glrlm_LongRunHighGrayLevelEmphasis_t2, left accumbens area, right accumbens area |
| IDH Status | Spatial distribution pattern | left thalamus, left putamen, left ventral DC, right thalamus, right caudate |
|  | Radiomics | firstorder_Median_t1, original_gldm_DependenceVariance_t1, shape_Maximum2DDiameterRow_t1ce, shape_MeshVolume_t1ce, firstorder_RootMeanSquared_flair, firstorder_Skewness_flair, glcm_Idmn_flair, original_glcm_InverseVariance_flair, gldm_DependenceNonUniformity_flair, gldm_LargeDependenceLowGrayLevelEmphasis_flair |
|  | Combined | firstorder_90Percentile_t1, original_firstorder_InterquartileRange_t1, glcm_JointEntropy_t1, original_shape_SurfaceArea_t1ce, gldm_DependenceNonUniformityNormalized_t1ce, firstorder_90Percentile_flair, firstorder_Uniformity_flair, glcm_ClusterTendency_flair, glrlm_HighGrayLevelRunEmphasis_t2, left putamen |
| Overall Survival Status | Spatial distribution pattern | left hippocampus, right cerebral white matter, right cerebral cortex, right pallidum, right amygdala |
|  | Radiomics | firstorder_Median_t1, glcm_Imc2_t1, glszm_HighGrayLevelZoneEmphasis_t1, glszm_SizeZoneNonUniformity_t1, glszm_SmallAreaHighGrayLevelEmphasis_t1, glszm_GrayLevelNonUniformity_t1ce, glrlm_ShortRunLowGrayLevelEmphasis_flair, glrlm_LongRunLowGrayLevelEmphasis_t2, glszm_SmallAreaEmphasis_t2, gldm_GrayLevelNonUniformity_t2 |
|  | Combined | glszm_SizeZoneNonUniformity_t1, gldm_SmallDependenceEmphasis_t1, glcm_ClusterShade_t1ce, glrlm_RunLengthNonUniformity_t1ce, firstorder_Uniformity_flair, glcm_Imc2_t2, glrlm_LongRunLowGrayLevelEmphasis_t2, 3rd ventricle, left amygdala, right thalamus |

Table 6. Statistical comparison of the quantified AUROC metrics between the best performing models.

| **Comparison** | **Task** | **Dataset** | | | |
| --- | --- | --- | --- | --- | --- |
|  |  | RSNA | UPenn | UCSF | LUMIERE |
| Spatial vs. Radiomics | MGMT | 0.0295 | 0.0183 | $<$0.0001 | 0.0024 |
|  | IDH | NA | 0.0158 | $<$0.0001 | 0.0073 |
|  | Survival | NA | 0.0089 | 0.0146 | 0.0009 |
| Radiomics vs. Combined | MGMT | 0.0771 | 0.0362 | 0.0678 | 0.0328 |
|  | IDH | NA | 0.0773 | $<$0.0001 | $<$0.0001 |
|  | Survival | NA | 0.0312 | 0.0509 | 0.0046 |

Table 7. Reported performance metrics for MGMT Methylation status prediction in the literature

| SOTA MGMT Methylation Performance | | |
| --- | --- | --- |
| **Author (reference)** | **Dataset** | **Reported AUROC** |
| *Calabrese et al. (36)* | Private, 191 structural, diffusion, perfusion MRIs | 0.55 |
| *Saeed et al. (37)* | RSNA | 0.630 |
| *Capuozzo et al. (38)* | RSNA | 0.598 |
| *Emchinov et al. (40)* | RSNA | 0.650 |
| *Ablet et al. (15)* | RSNA | 0.620 |
| *Korkmaz et al. (41)* | Subset of UCSF | 0.890 |
| *Chen et al. (42)* | Private, 111 structural and diffusion MRIs | 0.91 |

1. https://surfer.nmr.mgh.harvard.edu/ [↑](#footnote-ref-1)
